# The Role of Pet-Based Activities: Working with Rabbits to Improve Self-Esteem in Preschool Children

**DOI:** 10.3390/ani14243565

**Published:** 2024-12-10

**Authors:** Sang-Hwan Kim

**Affiliations:** 1 School of Animal Life Science, Hankyong National University, 327, Jungang-ro, Anseong-si 17579, Gyeongggi-do, Republic of Korea; immunoking@hknu.ac.kr; Tel.: +82-31-670-5127; 2 Institute of Applied Humanimal Science, Hankyong National University, 327, Jungang-ro, Anseong-si 17579, Gyeonggi-do, Republic of Korea

**Keywords:** animal-mediated therapy, animal-assisted activity, self-esteem, social science, rabbit-assisted activities, children

## Abstract

Animal-assisted activities (AAAs) effectively improve the self-esteem of infants and young children, thereby playing a significant role in their growth into adolescence. In particular, in modern society, self-esteem during childhood and adolescence can influence self-esteem during adulthood. Therefore, this study evaluates whether animal-assisted activities could improve infants’ self-esteem. There were ten participants in this study, with six one-hour sessions conducted twice a week with six-year-old children. The study proceeded with one host, two assistant hosts, one animal manager, one observer, and two helper rabbits. A self-esteem test was used as the research tool. Before and after the program, the pre-and post-tests were conducted separately to confirm the effect. Animal-mediated therapy in the program resulted in reducing the average scores in certain sub-areas of self-esteem. Additionally, the average score demonstrated a statistically significant increase in other areas. This study’s results showed that play programs involving animal-assisted activities can positively improve the self-esteem of infants and toddlers who develop early social skills.

## Simple Summary

Six-year-old children who are not socially developed generally show low self-esteem, but this level increases continuously due to teacher interaction. However, using methods such as animal-assisted activities incorporating animals can rapidly increase the levels of self-esteem in children. This study investigated the effectiveness of rabbit-based intervention in two groups with similar backgrounds. It can be concluded that rabbit-based intervention is particularly effective in increasing children’s self-esteem. In addition, self-esteem was effectively improved in boys compared to girls. This method helped improve children’s early sociality and self-esteem.

## 1. Introduction

Internationally, low birth rates have led to a decline in the formation of concepts related to children’s behavior and self-esteem. In Korea, a predominantly child-centered society, parents frequently influence the formation of self-esteem during children’s emotional development. According to the current survey on the happiness index of children and adolescents, Korea obtained the 22nd rank out of 22 countries for “subjective happiness”, and its index was the lowest among Organisation for Economic Co-operation (OECD) countries [1]. Among the detailed indicators, subjective health and life satisfaction ranked 22nd out of the 22 OECD countries (Key Member Countries: the United States, Switzerland, Norway, Luxembourg, Denmark, Iceland, Sweden, Germany, Ireland, the Netherlands, Austria, Australia, Canada, Belgium, France, Finland, Japan, the United Kingdom, New Zealand, Italy, Spain, and South Korea), whereas loneliness ranked 21st. Notably, the “subjective happiness index” decreased by nine points compared what it was to two years ago. According to an analysis of the Child Happiness Index conducted by the Green Umbrella Children’s Foundation, the number of surveys on impulsive suicidal thoughts increased significantly from 2021 to 2023. Of all the children, about 10.2% responded that they had impulsive suicidal thoughts. These results are reflected in the increases in scores for neglect and emotional abuse by guardians, depression, and anxiety; consequently, self-esteem is on a declining trend. These statistical indicators suggest that social and environmental factors are causing self-esteem among growing children to decline [2,3]. According to Baldwin [4], developing self-esteem is an important part of childhood and adolescence and is essential for survival, especially for young children. Early childhood self-esteem is defined as the belief that toddlers and preschoolers can excel and perform better than others in any field [5]. Additionally, early childhood self-esteem refers to respect for oneself, positive or negative evaluations, and an attitude toward one’s values [6].

Various studies have examined the relationship between children’s happiness and self-esteem and have found that the latter has a significant positive impact on the former. In particular, when comparing peer children, the higher the self-esteem, the higher the happiness; conversely, as self-esteem declines, so does happiness, at the same rate [7]. This suggests that changes in self-esteem may be correlated with positive changes in happiness [6]. These results indicate the need for mental health services that promote the development of children’s emotions and self-esteem.

Animal-assisted therapy (AAT) has recently gained attention as a complementary and alternative medicine and is known to improve physical and mental well-being by utilizing the bond between humans and animals. Pet-centric psychotherapy, developed by Boris Levinson [8,9], began with experiments conducted on hospital patients, with the use of pets, by Samuel and Elizabeth Corson. They confirmed improvements in self-esteem, responsibility, and social interaction [10]. Since then, in the 1980s, more research has been conducted in various fields, such as veterinary medicine, psychology, and social work, and the use of animals to help people relax and reduce anxiety has gained popularity [11].

AAA is a new form of AAT that, unlike AAT, does not include psychological treatment and believes that children can approach problem-solving independently through simple arts and crafts play with animals [12]. In particular, AAA is thought to be able to influence children’s self-development without utilizing active psychological treatment.

Thus, animal-assisted therapy can stimulate human emotions to induce positive emotions, and this effect may be more significant in young children. This study aimed to confirm whether animal-assisted activities (AAAs) affect the development of self-esteem and positive emotions in preschool children in Korea.

## 2. Materials and Methods

### 2.1. Ethical Approval for Animals and Humans

The study was conducted according to the guidelines of the Declaration of Helsinki and was exempted by the Institutional Animal Care and Use Committee Ethics Committee of Hankyong National University (protocol code 2024-1, exempted on 22 October 2024) for the use of animals. For the children-related portion, ethical review and approval were waived under Article 13 of the Enforcement Decree of the Bioethics and Safety Act, as this activity is not animal-assisted therapy (AAT) but animal-assisted activity (AAA) and is therefore not intended for psychotherapy concerning the children who are the subjects of the activity.

### 2.2. Participants

Two local daycare centers that were previously affiliated with universities were selected for children’s participation. Twenty children aged 6 years old who were learning at each daycare center were selected and allowed to participate in the program after obtaining written consent from their parents. Twenty children and their parents were allowed to participate in the animal-assisted activity after they were informed about the safety and efficacy of the “best practices” of conducting psychosocial interventions using animals.

In order to compare the changes in self-esteem due to animal-assisted activities, children were randomly assigned to two groups, AAA and non-AAA, with 10 children in each. One group participated in animal-assisted activities (AAAs) with rabbits, and the non-AAA group participated in animal-assisted activities without rabbits, observing rabbits only through videos and pictures. The experimental and control groups comprised 5 boys and 5 girls. However, in the analysis of the results of this study, differences between boys and girls in the experimental group were analyzed only, and no comparison was made with the control group because the analysis focused on significant differences between boys and girls in the experimental group.

### 2.3. Inclusion and Exclusion Criteria

Participants were selected using a multiple screening procedure for parents, teachers, and participating children. To objectively assess the possibility of inclusion and exclusion from children’s activities before participation, we analyzed the children’s status before participation using the Rosenberg Self-Esteem Scale and the Korean Child Behavior Checklist [13,14,15]. The inclusion criteria included a normal psychological state and very little contact with animals. The exclusion criteria included current psychological or behavioral problems; a diagnosis of pervasive developmental disorder/autism, depression, anxiety, or epilepsy; and a history of animal abuse.

Children were included or excluded based on each criterion, and the test results on the possibility of participation were destroyed at the institution’s request. After that, only the self-esteem test developed by Coopersmith was used to analyze animal-assisted activities.

### 2.4. Training of AAA Facilitators

The AAA facilitators were college students majoring in animal life sciences, with a certificate accredited by the Association of Korean Companion Animal-Assisted Therapy, who had performed AAAs in a local university city for a certain period. After 45 min of cognitive meetings, direct physical contact (e.g., stroking) was induced. In the first training session, rules and regulations regarding animal welfare and human behavior toward rabbits were explained, and animal-assisted cognitive training was conducted in groups using various methods. Any shortcomings (movement, vision, hearing, social communication skills, and touch) improved with the exercise.

### 2.5. Selecting Animals for AAAs

One-year-old rabbits participated in AAAs. Rabbits are generally popular with children because of their characteristics of easy socialization, friendly behavior, and clear gestures [16,17]. Additionally, it was believed to be a good opportunity to stimulate caution and curiosity that arises among children from contact with animals smaller than themselves.

### 2.6. Rabbit Welfare for Animal-Mediated Activities

All rabbits were assessed for health before the activity, vaccinated against myxomiasis and rabbit hemorrhagic disease, and treated for parasites. The rabbits that participated in this study were free of disease or parasites. Feces were continuously monitored to identify health problems [16,18,19]. The rabbits were fed a complete diet of pellet food, which is rich in fiber for good intestinal function, and selective feeding was prevented. Additionally, hay and fresh vegetables were provided daily. Food was provided twice daily (in the morning and at the end of the activity), and hutches were cleaned and replaced with dust-free bedding by the participating children. Water was available ad libitum from a teat drinker, and hay was continuously available from a hay rack. Gnawing sticks were used to enrich the environment, and mineral supplement blocks were installed in the hutch. Following their daily routine, the rabbits remained calm in the hutch and consumed food and water. When the hutches were placed on the play carpet, they came out of them, walked around the classroom, and were petted by the children. Over time, when they became tired, they returned to their hutches, which the children always respected.

### 2.7. Organizing an Animal-Assisted Activity Program

The program for AAA was conducted according to the manual provided by the Association of Korean Companion Animal-Assisted Therapy [12,20]. The validity of rabbit-assisted activities and the evaluation of children’s voluntary interactions were based on the results of a study by Molnár [16]. Based on the analysis results according to the children’s selection conditions, the following program was created and implemented:

In the program’s first session, “Hello, Friend!”, first the counselors, and then the helper animals, were introduced. The species, name, age, gender, and characteristics of the helper animals were explained, and a greeting was initiated. Subsequently, a name tag was created to introduce the participant to the program. After name tags were assigned and introductions were made, quiet time was spent, with participants making eye contact with the animal friends. The expected effects included building rapport with the animal, gaining a sense of accomplishment and satisfaction, and improving self-esteem.

The second session, “Decorate Your Own Animal Friend Picture”, involved attaching an OHP film to a side photo of the helper animal and tracing the shape with an oil-based marker. Subsequently, only the film was removed and attached to a hardboard, and a handle was made by attaching a fur wire to the hardboard. The expected effects included building rapport with the helper animal, improving expressiveness, gaining a sense of accomplishment, and significantly boosting self-esteem.

The third session, “Decorate the Animal Friend Tableware Set”, explained that helper animals, similar to people, need tableware. A food bowl was made for the helper animals. The completed dish was filled with food and water in order for the helper animal to eat/drink from it. The expected effects were improving self-esteem and maximizing the sense of accomplishment.

In the fourth session, “I will Make Something Delicious”, the children learned about foods that are good and those that are unsuitable for their animal friends. Next, food suitable for the animal friend and the children’s favorite foods were made from clay and exchanged for real snacks. The expected effects were improving self-esteem, stimulating tactile sensations, and gaining a sense of accomplishment.

In the fifth session, “Mission Game with Animal Friends”, the children answered the O/X quiz about the animals and collected stickers. The quiz tested the obtained information about helper animals gained during activity periods and through interactions with them. The expected effects were a strong sense of cooperation, feelings of accomplishment, intimacy through skinship, and improved self-esteem.

Finally, in the sixth session, “Thank You, Friend!”, memory notes were created, working with photographs from the previous program. The emotions and thoughts of each session were expressed through drawings and writing. The participants were to talk with their counselor about what they had learned and their memories. The expected effects were improvements in self-esteem, self-resilience, and sociality.

### 2.8. Self-Esteem Change Analysis

The self-esteem scale was modified and supplemented for use with infants and toddlers, based on a self-esteem test developed by Coopersmith [21,22,23] and translated into Korean by Chio and Jeon [24]. The questions comprised four subscales: “Overall Self-Esteem”, “Social Self-Esteem”, “Family Self-Esteem”, and “School Self-Esteem”. There were 32 questions, 15 of which were appropriate for infants and toddlers. This self-report scale measures attitude. Each question was scored as 1 point for “far from it”, 2 points for “but no”, 3 points for “average”, 4 points for “agree”, and 5 points for “very much agree”. Questions 1, 5, 12, and 15 pertained to overall self-esteem; questions, 2, 6, 9, and 13 pertained to social self-esteem; questions 3, 7, 10, and 14 related to family self-esteem; and 4, 8, and 11 related to school self-esteem. Questions 4, 8, and 11 were scored in reverse (Table 1). The total possible score ranged from 15 to 75, with higher scores indicating higher (positive) self-esteem: 15 to 34 points indicated low self-esteem; 35 to 54 points indicated average self-esteem; 55 points indicated healthy and desirable self-esteem. The self-esteem scale was analyzed as a pre-test before the start of the animal-assisted activity and as a post-test after all the animal-assisted activities were completed. The same testing method was applied to the control group as well.

### 2.9. Ethical Considerations

This study was conducted with the consent of the parents and children (participants). They were informed that the process would be filmed and recorded for research purposes only and that confidentiality of identity would be guaranteed. Additionally, participation could be withdrawn midway in case of any inconvenience during participation. Only children who agreed to participate in the program were included. Pre-trained facilitators participated to ensure the safety of the activity.

### 2.10. Statistical Analysis

The scores for effects on children’s self-esteem were analyzed using a generalized linear model (GLM) analysis, considering that the same children were assessed repeatedly. Statistical analyses were performed using IBM SPSS 21.0.0.0 software (IBM, SPSS Inc., Endicott, NY, USA).

## 3. Results

### 3.1. Pre- and Post-Results of Self-Esteem in the Group with AAAs

The group undergoing the AAA program was told that they would do an animal-mediated activity with rabbits, and the non-AAA group was told that they would do a fun activity today, providing hints to increase interest, and then a self-esteem test was administered.

The overall self-esteem evaluation before and after AAA generally revealed positive effects, with changes ranging from a maximum of 12 points to a minimum of 1 point. In the cases of two participants, negative elements were formed in the post-evaluation results, but there was no significant difference, with a score of −1 point (Table 2). There were differences in self-esteem between boys and girls, with significant changes generally observed among boys (Table 2). In the case of the control group children who participated in non-AAAs, there was no significant difference because they participated in the program they usually did, and in general, there was a change of at least 5 points from a maximum of 9 points. The average difference between AAA and non-AAA was not high. However, there were relatively various changes in AAA compared to non-AAA.

### 3.2. Changes in Overall Self-Esteem Between the Control Group and the Activity Participation Group

The overall average score changes in self-esteem for total AAA increased significantly by about 6 points from the pre-test (54.85714) to the post-test (60.14286) in the experimental group. However, in the control group, which was the normal group, it increased by approximately 8 points, demonstrating a higher trend than that in the experimental group (Table 3).

### 3.3. Comparative Analysis of Changes in Children’s Overall Self-Esteem

The overall self-esteem change trend, which determines the detailed characteristics of self-esteem, revealed a significant change of 2 points from the pre-test (15.28571) to the post-test (17.28571) in the experimental group. In contrast, the control group showed an improvement of approximately 1 point from the pre-test (15.33333) to the post-test (16), thereby showing better results among the experimental group (Table 4).

### 3.4. Analysis of Changes in Socially Connected Self-Esteem

**Figure 1:**
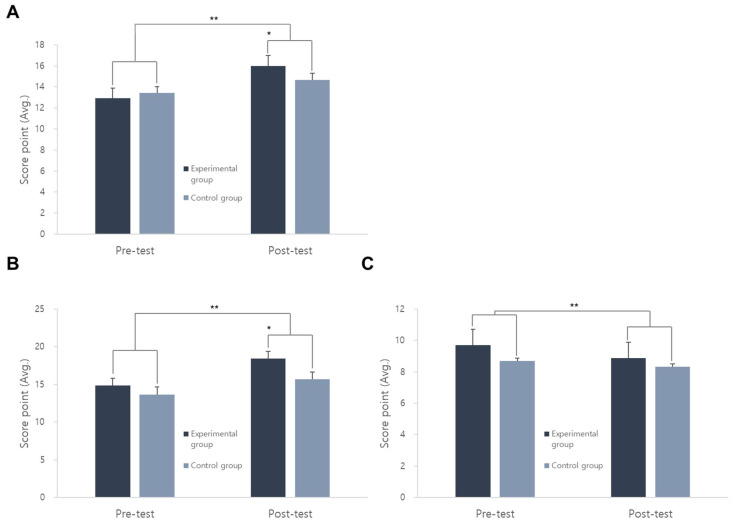
Analysis of changes between the control group and the experimental group according to self-esteem sub-items. (**A**) Social self-esteem test; (**B**) Family self-esteem test; (**C**) Self-esteem in school test. *, ** indicate significant differences (*p* < 0.05).

**Figure 2:**
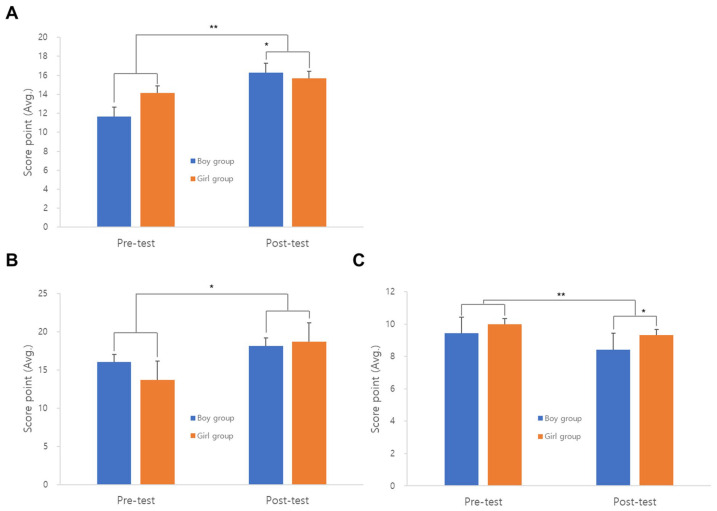
Analysis of changes between girls and boys according to self-esteem subscales. (**A**) Social self-esteem test; (**B**) Family self-esteem test; (**C**) Self-esteem in school test. *, ** indicate significant differences (*p* < 0.05).

In the analysis of self-esteem, the experimental group increased overall compared to the control group. In the analysis of change trends by detailed items, the family self-esteem of the experimental group increased from 12.904 to 15.976 (Figure 1A). In particular, the evaluation of social self-esteem significantly increased from 14.833 to 18.404 in the experimental group compared to the control group (Figure 1B), and school self-esteem decreased by about 1 point in the experimental group, but there was no change in the control group (Figure 1C). The overall comparison showed that the experimental group significantly increased compared to the control group. Overall, social self-esteem in the boy group increased from 14.142 to 16.285 (Figure 2A). However, between the pre- and post-tests, family self-esteem increased from 13.666 to 17.666 in the girl group (Figure 2B); school self-esteem decreased from 9.6 to 8.7 in the boy group (Figure 2C).

In the girl group, there was a significant increase in overall self-esteem after the activity, but it was lower than that in the boy group. Among the elements of self-esteem, we believe that the results for family self-esteem were positive because at the end of the activity, phrases related to the family, such as “let us go home and brag to our family” and “let us talk about what happened today with our family”, were used. Afterward, during the activity, participants could be heard saying, “I will show my mom what I made today”, “Mom and dad said that my rabbit is cool”, and so on. It appears that family self-esteem was influenced by the time spent talking about AAA with families after each activity.

## 4. Discussion

Animal-mediated psychological therapy influences children’s emotional development and cognitive enhancement, particularly in recovering self-esteem affected by parental social constraints [25,26]. Animal-mediated psychological therapy positively affects social awareness and social members, and the presence and value of living animals can increase the need for self-regulation and awareness [26,27,28]. This study analyzed whether AAA during children’s developmental stages could influence their self-esteem and enhance self-awareness. According to Molnár [16], working with rabbits in mediated activities resulted in increasing the overall self-esteem development of the children who participated in the activities, as compared to those who did not. In particular, children’s social self-esteem increased positively in all activities compared with the non-participating group, strengthening their motivation to participate [4,28]. Several studies have shown that involving therapeutic animals in activities reduces children’s overall social stress and increases their motivation for academic achievement. Animal-assisted interventions positively affect motivation for social participation, self-efficacy, attention, self-control, and group-related competencies [29].

Additionally, working with small animals, such as rabbits, in interventions can increase interactions between animals and humans, although it may not significantly impact children’s boldness, as shown by Molnár [16]. This suggests that small animals, such as rabbits, can be incorporative for children’s social cognitive development and that the core elements of self-esteem can be improved through AAA. These results showed significant differences between the two groups (non-animal-assisted activity group/animal-assisted activity group), confirming that interactions with living animals affected children’s behavior positively.

The decrease in school self-esteem from the pre-test (9.428571) to the post-test (8.428571) is believed to be influenced by psychological factors, such as participants demonstrating negative reactions or tears when told, “This is the last session, and we will not see each other again”. Additionally, some participants’ scores decreased when they expressed disappointment in response to the question, “Is there anything disappointing at the daycare center?” The short six-session program may not have allowed sufficient time for the termination phase. Similar results were found by Kim [30,31], where animal-assisted therapy programs for lower-grade elementary school children with withdrawn behavior increased overall self-esteem, detailed overall self-esteem, social self-esteem, and family self-esteem, but decreased school self-esteem.

In conclusion, the changes in children’s self-esteem scores across various areas of the study significantly increased in the AAA group compared to the non-AAA group, suggesting that these changes may be due to animal intervention. Most children participating in this study evaluated their abilities more favorably than their peers of the same age. This finding suggests that changes in self-awareness and social self-esteem are influenced, and motivation to participate in activities increases with animal intervention [31,32].

In particular, our study analyzed the effects of activities with/without rabbit intervention on children’s self-esteem and confirmed that AAAs with rabbit intervention had a positive effect on the experimental group compared to the control group. These results suggest that AAAs without psychotherapy can also have a positive effect on improving children’s self-esteem.

This study has some limitations. First, it is difficult to generalize the results to all children because of the small sample size of 10 participants in the AAAs. Second, establishing children’s growth environments was challenging. Third, the short six-session program made it difficult to predict changes in children’s self-esteem after long-term AAAs. However, the changes in self-esteem observed in children during the short six-session program suggest that AAAs can help enhance the value of animals and humans and positively impact the development of positive self-esteem.

Animal-assisted activities (AAAs) are generally expected to have lower or temporary effects compared to animal-assisted therapy combined with psychotherapy. However, our research has shown that there are at least partial psychological changes in children through participation in AAAs. Therefore, based on our research, if we can continuously develop customized programs for these subjects, develop standardized assessment tools to increase the reliability of the research results, and build a system that can analyze them from various angles, we will be able to develop and utilize more effective programs.

## 5. Conclusions

Our study analyzed whether AAAs working with small animals, such as rabbits, could affect self-esteem in children’s developmental stages and confirmed a significant increase in children’s self-esteem in the AAA-group, compared with those in the non-AAA group. The beneficial effects of AAAs showed that the initial anxiety decreased with interactions with animals, positively affecting social cognition. The involvement of rabbits in AAAs was suitable for improving children’s educational activities. In particular, teachers’ evaluations after the program indicated positive effects on social participation, as they said that “Children spoke confidently and proactively participated in activities”, “Even shy children asked many questions, improving their participation”, and so forth. Therefore, AAAs may reduce children’s anxiety levels and positively influence their self-esteem.

## Figures and Tables

**Table 1:** Questions of self-esteem test.

Subscales	Questions Number	Number of Questions
Total	1, 5, 12, 15	4
Social	2, 6, 9, 13	4
Family	3, 7, 10, 14	4
School	4 *, 8 *, 11 *	3
Total questions		15

* Reverse scoring questions.

**Table 2:** Questions of self-esteem test.

Experimental Group					Control Group				
NO	Sex	Pre	Post	Results	NO	Sex	Pre	Post	Results
1	Boy	58	68	+10	1	Boy	52	60	+8
2	Boy	56	67	+11	2	Boy	53	61	+8
3	Boy	57	62	+5	3	Boy	55	63	+8
4	Boy	55	67	+12	4	Boy	49	58	+9
8	Boy	42	50	+8	8	Boy	55	60	+5
6	Girl	48	47	−1	6	Girl	54	63	+9
7	Girl	44	54	+10	7	Girl	55	63	+8
5	Girl	70	71	+1	5	Girl	47	55	+8
9	Girl	56	55	−1	9	Girl	46	55	+9
10	Girl	53	59	+6	10	Girl	50	58	+8

**Table 3:** The effect of animal-assisted activities on overall questions of self-esteem (*p* < 0.05).

	Pre-Test	Post-Test	Paired t	*p*-Value
	M (SD)	M (SD)		
Experimental group	54.85714 (1.25487)	60.14286 (1.80206)	−1.93949	0.020058
Control group	51.66667 (1.3865)	59.66667 (1.73689)	−4.33333	0.0284171

**Table 4:** The effect of animal-assisted activities on total self-esteem (*p* < 0.05).

Total Self-Esteem	Pre-Test	Post-Test	Paired t	*p*-Value
	M (SD)	M (SD)		
Experimental group	15.28571 (0.81154)	17.28571 (0.09377)	−2.44949	0.0114809
Control group	15.33333 (0.16333)	16 (0.60555)	0.666667	0.0337779

## Data Availability

The original contributions presented in the study are included in the article material, further inquiries can be directed to the corresponding author.

## References

[1] Yeom Y.S. (2022). Korean Children and Youth Well-Being Index Survey, 2021: Elementary School Students.

[2] Diener M.B., Milich R. (1997). Effects of positive feedback on the social interactions of boys with attention deficit hyperactivity disorder: A test of the self-protective hypothesis. J. Clin. Child Psychol..

[3] Hoza B., Vaughn A., Waschbusch D.A., Murray-Close D., McCabe G. (2012). Can children with ADHD be motivated to reduce bias in self-reports of competence?. J. Consult. Clin. Psychol..

[4] Baldwin S.A., Hoffmann J.P. (2002). The Dynamics of Self-Esteem: A Growth-Curve Analysis. J. Youth Adolesc..

[5] Harter S., Leary M.R., Tangney J.P. (2003). The development of self-representations during childhood and adolescence. Handbook of Self and Identity.

[6] Choi N.R., Cho H.J. (2014). The effect of young children’s emotional intelligence and self-esteem on their happiness. J. Learn.-Cent. Curric. Instr..

[7] Lee J.K., Cho H.C. (2012). A Longitudinal Study of Factors Associated with Happiness in Primary School Children. J. Korean Soc. Child Welf..

[8] Havener L., Gentes L., Thaler B., Megel M.E., Baun M.M., Driscoll F.A. (2001). The effects of a companion animal on distress in children undergoing dental procedures. Issues Compr. Pediatr. Nurs..

[9] Fine A.H. (2005). Handbook on Animal-Assisted Therapy: Foundations and Guidelines for Animal-Assisted Interventions.

[10] Corson S.A., Corson E.O., Gwynne P.H., Arnold L.E. (1975). Pet-facilitated psychotherapy in a hospital setting. Curr. Psychiatr. Ther..

[11] Grandgeorge M., Hausberger M. (2011). Human-animal relationships: From daily life to animal-assisted therapies. Ann. Ist. Super. Sanit..

[12] Kim B.T., Kim S.H., Kim K.W., Park Y.S., Jin M.R. (2021). Companion Animal Therapy.

[13] Rosenberg M. (1965). Society and the Adolescent Self-Image.

[14] Rosenberg M. (1989). Society and the Adolescent Self-Image.

[15] Park E.Y., Seo H., Blair K.S.C., Kang M.C. (2021). Rasch Analysis of the Korean-Child Behavior Checklist (K-CBCL) to Assess Emotional and Behavioral Problems in Children with Autism Spectrum Disorder. Sage Open.

[16] Molnár M., Iváncsik R., DiBlasio B., Nagy I. (2019). Examining the Effects of Rabbit-Assisted Interventions in the Classroom Environment. Animals.

[17] Mallon G.P. (1992). Utilization of animals as therapeutic adjuncts with children and youth: A review of the literature. Child Youth Care Forum.

[18] Molnár M., Takács I., Rudolf Z., Molnár T. Examination the confidentiality of rabbits involved in animal-assisted pedagogical work. Proceedings of the Conference of the Hungarian Ethological Society.

[19] Walter-Toews D. (1993). Zoonotic disease concerns in animal-assistedtherapy and animal visitation programs. Can. Vet. J..

[20] Kang W.G., Jang S.M., Kim O.J. (2018). Study on Animal-assisted Therapy Regarding Self-esteem and Sociality of Children from Low-income Families in Rural Areas. J. Rural Soc..

[21] Coopersmith S. (1987). Self-Esteem Inventories.

[22] Coopersmith S. (2002). Revised Coopersmith Self-Esteem Inventory Manual.

[23] Choi B.G., Jeon G.Y. (1993). Study on the Development of the Self-Esteem Inventory. Int. J. Hum. Ecol..

[24] Park H.J., Kim C.H. (2012). The effects of an animal-assisted therapy (AAT) program on depression and self-esteem of adolescents as victims of school violence. Korean J. Vet. Serv..

[25] Lee Y.J. (2022). Effects of Animal-Assisted Counselling on Self Esteem and Emotive Behavior of Children. J. Korean Assoc. Anim. Assist. Psychother..

[26] Schuck S.E.B., Emmerson N.A., Abdullah M.M., Fine A.H., Stehli A., Lakes K.D. (2018). A randomized controlled trial of traditional psychosocial and canine-assisted intervention for children with ADHD. Hum.-Anim. Interact. Bull..

[27] Schuck S.E.B., Johnson H.L., Abdullah M.M., Stehli A., Fine A.H., Lakes K.D. (2018). The Role of Animal-Assisted Intervention on Improving Self-Esteem in Children With Attention Deficit/Hyperactivity Disorder. Front. Pediatr..

[28] Gee N.R., Mueller M.K., Curl A.L. (2017). Human-Animal Interaction and Older Adults: An Overview. Front. Psychol..

[29] Kim J.Y., Kang W.G., Lee Y.S., Shin S.H., Kim M.S., Kim S.B., Lee S.Y., Jung S.U., Hwang H.J., Kim O.J. (2018). Effects of Animal-assisted Therapy on Self-esteem and Peer Relationship in Lower Grade Elementary School Children with Atrophy. J. Anim. Assist. Psychother..

[30] Kim S.W. (2022). Examining the Relationship between Happiness and Self-esteem Using Autoregressive Cross-lagged Modeling. Korean Soc. Wellness.

[31] Hediger K., Meisser A., Zinsstag J. (2019). A One Health Research Framework for Animal-Assisted Interventions. Int. J. Environ. Res. Public Health.

[32] Kim K.H. (2020). Childhood Loss and Depressive Symptoms in Adulthood: The Mediating Effect of Positive Relations. Hallym J. Aging Stud..

